# Unprompted Closure of Spontaneous Macular Hole Secondary to Retained Subfoveal Perfluorocarbon Liquid

**DOI:** 10.1155/2022/5306029

**Published:** 2022-09-26

**Authors:** Hamouda Hamdy Ghoraba, Hashem Hamouda Ghoraba, Adel Galal Zaky

**Affiliations:** ^1^Ophthalmology Department, Faculty of Medicine, Tanta University, Tanta, Egypt; ^2^Magrabi Eye Hospital, Tanta, Egypt; ^3^Ophthalmology Department, Faculty of Medicine, Menoufia University, Shibin el Kom, Egypt

## Abstract

**Purpose:**

To report unprompted closure of spontaneous macular hole secondary to inadvertent subfoveal perfluorocarbon liquid (PFCL) after vitrectomy surgery. *Observations*. We present a case of a retained large single subfoveal PFCL droplet following vitrectomy and silicone oil injection for subtotal rhegmatogenous retinal detachment that showed spontaneous release 3 weeks postoperatively, with subsequent development of full thickness macular hole (FTMH) which completely closed later on after silicone oil removal without internal limiting membrane peeling with marvelous anatomic and visual improvement after spontaneous closure of MH.

**Conclusions:**

Different fates of subfoveal PFCL droplets may happen. Spontaneous release of subfoveal PFCL without surgical interference has rarely been reported.

## 1. Introduction

The unique chemical and physical properties of perfluorocarbon liquid (PFCL) have enabled internal displacement of subretinal fluid and stabilization of mobile retina, transforming the surgical approach of complex retinal detachments repair. Despite these advantages, the use of PFCL may be complicated by inadvertent retention of PFCL droplets in the subretinal space [1]. Subfoveal PFCL may be visually significant, and prompt surgical removal or displacement is indicated according to the visual prognosis [1]. While peripheral subretinal PFCL can often be observed, migration of peripherally located droplets towards the fovea is well recognized, although the mechanisms of migration remain unknown [2]. Spontaneous resolution of subfoveal PFCL without surgical evacuation or displacement has rarely been described [3]. We present a case of a retained large single subfoveal PFCL droplet that spontaneously resolved in out of classic manner.

## 2. Case Report

**Figure 1:**
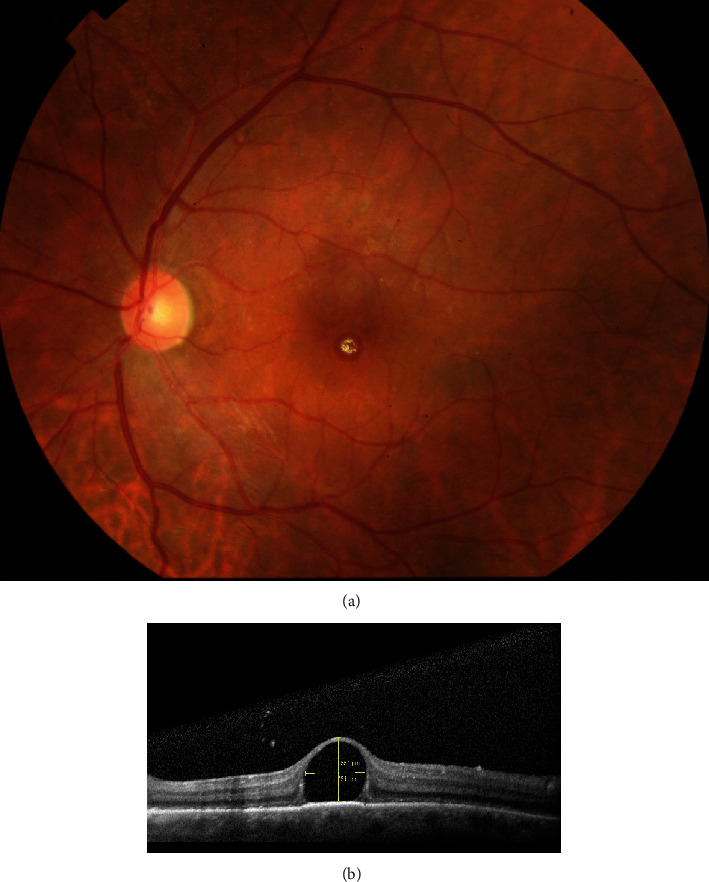
(a) Colored fundus photography showing subfoveal PFCL droplet. (b) OCT showing Ώ sign.

**Figure 2:**
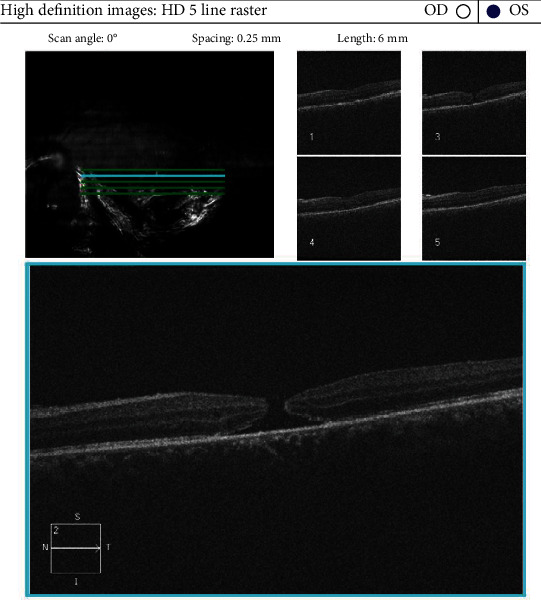
OCT showing development of FTMH.

**Figure 3:**
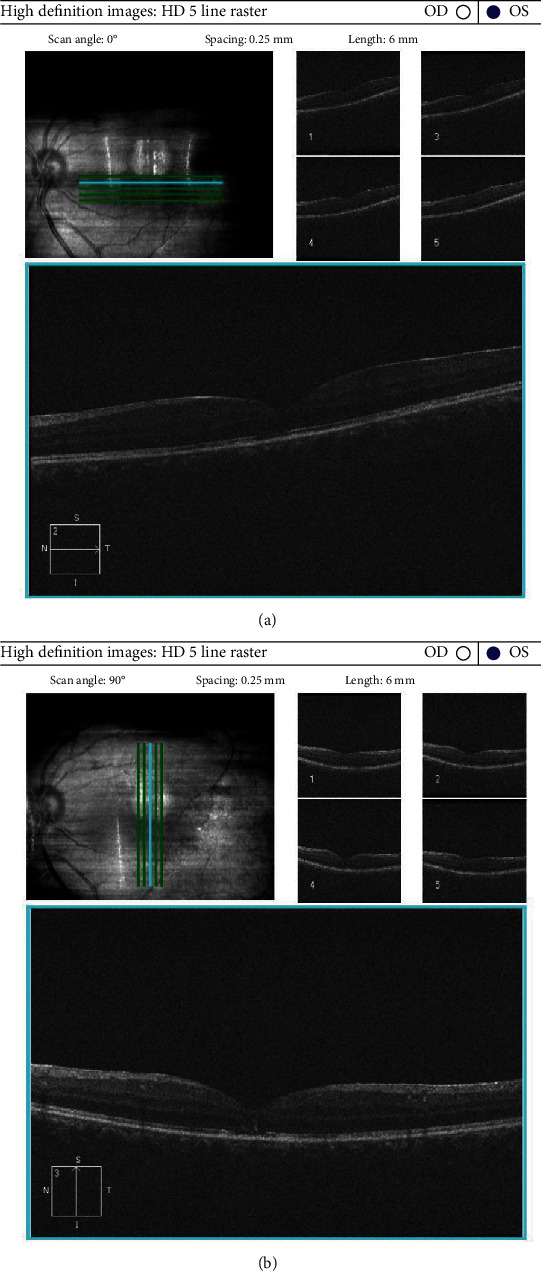
OCT horizontal and vertical HD raster scan showing closure of MH with improvement of microarchitecture of the retina.

A male patient, 52 years old, presented with subtotal rhegmatogenous retinal detachment with single large upper equatorial break (10-12 o' clock) with macula off in the left eye with visual acuity of hand movement. He underwent pars plana vitrectomy (PPV) and silicone oil injection. Transconjunctival 23 gauge 3-port PPV technique using the EVA surgical system (Dutch Ophthalmic Research Center (DORC), Zuidland, Netherlands) and a noncontact wide-angle viewing system BIOM (binocular indirect ophthalmoscope; Oculus, Wetzlar, Germany) were used. Posterior vitreous detachment was created using aspiration by a vitreous cutter with triamcinolone. PFCL was used to stabilize the posterior pole, and the vitreous base was shaved 360 degrees as safe as possible without lensectomy using high-speed vitreous cut rates (8000-9000 cuts/min) and low vacuum settings (100-150 mmHg). Conventional internal limiting membrane (ILM) peeling was performed after injection of brilliant blue dye (ILM Blue; DORC International, Zuidland, Netherlands) under PFCL. Subretinal fluid was drained anteriorly through the original break with the assistance of PFCL by injection with 23-gauge dual bore cannula (MedOne Surgical, Sarasota, USA) keeping the injection within the single PFCL bubble to avoid “satellite” bubbles followed by air-PFC exchange. Endolaser photocoagulation was performed around identifiable retinal tear. Silicon-air exchange using silicon oil 5000 centistokes was performed. On postoperative day 1 and then at postoperative week 1, the patient had counting finger vision with retinal attachment under silicone oil, but a subfoveal PFCL droplet was noted on ophthalmoscopy (Figure 1(a)) and confirmed with optical coherence tomography (OCT) imaging, which revealed a focal area of severely thinned retinal tissue overlying the droplet with omega sign (Figure 1(b)). After 2 weeks, visual acuity (VA) improved to 0.1, and the patient complained of metamorphopsia. The decision was made to aspirate PFCL by 41-gauge cannula after 1 week. The patient came at the time, but he complained of marked drop of vision with central scotoma; VA was counting fingers 2 meters. Fundus examination showed development of full thickness macular hole with disappearance of PFCL droplet which was confirmed by OCT (Figure 2). We preferred to follow the patient, and after 4 months from original surgery, he underwent phacoemulsification with intraocular lens implantation, and silicone oil removal (SOR) without any further ILM peeling techniques or free ILM flap and subretinal or intravitreal PFCL was never identified. In the 1st day and 1st week post, SOR fundus examination showed attached retina with FTMH, and VA was 0.05. On postoperative 3rd week, he noted improvement in his vision 3 days before the visit. By examination, VA was 0.4, and ophthalmoscopy was performed, demonstrating closure of MH. OCT performed that day revealed a restored foveal depression. Without any surgical intervention, these architectural changes improved over the subsequent months, with restoration of the ellipsoid zone at the fovea (Figure 3). VA improved to 0.7 after 3 months of SOR.

The study was approved by the Ethical Committee of the Magrabi Eye Hospital Foundation, and the patient was fully informed regarding the technique of the surgery, including the potential risks and complications, and signed a written informed consent document before surgery. This study followed the tenets and guidelines of the Declaration of Helsinki.

## 3. Discussion

Few complications have been associated with use of PFCLs. The most common is postoperative residual PFCL in vitreous cavity or in the anterior chamber. Subretinal retention of PFCL has been reported, and it has the most significant ocular toxicity. Inadvertent postoperative retention of PFCL occurs in approximately 1% to 7% of eyes. Multiple studies have shown that even with highly purified PFCLs, retinal toxicity occurs after just a few days of intravitreal PFCL exposure and an even shorter period for subretinal PFCL [2].

In this report, we demonstrate a case of spontaneous resolution of subfoveal PFCL. Examination of the posterior and peripheral retina just revealed FTMH without any subretinal PFCL droplets following release. We hypothesize that the surface tension of the PFCL droplet resulted in tangential forces that created a defect at the fovea which may be weakened by ILM peeling which was done in 1ry surgery. PFCL droplet is more likely to be spontaneously released, because the force acting to confine them in a normal retina might be disturbed [4]. We suggest that the PFCL droplet extruded through this retinal defect into the vitreous cavity, where it remained undetected, but was subsequently removed during SOR or may be allocated inferiorly at ciliary body. The possibility of retinal hole formation due to chronic subretinal PFCL retention with a very thin overlying retina has been reported in the literature.

Ghoraba et al. [5] revealed spontaneous formation of MHs in 2 patients. In those 2 patients, subfoveal PFCL droplets disappeared. Tanabu et al. [4] reported a case of MH secondary to subfoveal PFCL that subsequently closed after its spontaneous resolution. This happened after doing a vitrectomy for traumatic retinal detachment. They postulated that the retinal structure in the macula was fragile. Thus, subretinal PFCL droplets were likely to discharge more easily. Also, they explained that the eyes in which the ILM has already become detached or removed PFCL droplets are more likely to be spontaneously discharged.

In other studies, a large PFCL bubbles spontaneously migrated from the fovea in a downward direction or a subretinal PFCL droplet subsequently disappeared from the central fovea, resulting in improvement of the retinal structure [6]. Also, Oellers et al. [7] postulated that the PFCL droplet discharged through a transient hole created in the thinned retina overlying the droplet spontaneously, which later on closed leaving the macula flat and atrophic.

OCT was important especially for the follow-up of the patients and the differential diagnosis of retained subretinal fluid, subretinal SO, sticky SO, and subretinal PFCL because of their differences in viscosity, density, and surface tension. Entrapped PFCL bubble has distinct border, and retina adapts to the shape of Greek letter Ώ; thus, the angle between retinal pigment epithelium and neurosensory retina at base of PFCL bubble is acute, and retinal layers cannot be identified above PFCL droplet as whole retina was being squeezed with hyperreflective shadow at the choroid [5].

As regards VA, it was dramatically improved in our patient after release PFCL droplet and closure of MH and reached 0.7. This agrees with Oellers et al. [7] who reported in their case VA improvement to 20/70 at 6 weeks following oil removal, with sustained retinal attachment, absence of any retained PFCL, and continued improvement of the retinal contour and microarchitecture.

The mechanism of this rare event is scarcely reported in literature, and poorly recognized as different behaviors and scenarios of submacular PFCL droplets may occur [8]. In the future, improved understanding of the mechanisms of spontaneous resolution may enable the development of nonsurgical or minimal invasive techniques to remove subfoveal PFCL.

## Data Availability

The data sets used and/or analyzed during the current study are available from the corresponding author on reasonable request.
